# Human cranial vault thickness in a contemporary sample of 1097 autopsy cases: relation to body weight, stature, age, sex and ancestry

**DOI:** 10.1007/s00414-016-1324-5

**Published:** 2016-02-25

**Authors:** H. H. (Hans) De Boer, A. E. (Lida) Van der Merwe, V. (Vidija) Soerdjbalie-Maikoe

**Affiliations:** 1 Department of Pathology, Academic Medical Center, P.O. box 22660, Meibergdreef 9, 1100 DD Amsterdam, The Netherlands; 2 Department of Anatomy, Embryology and Physiology, Academic Medical Center, P.O. Box 22660, Meibergdreef 9, 1100 DD Amsterdam, The Netherlands; 3 Netherlands Forensic Institute, P.O. box 24044, Laan van Ypenburg 6, 2490 AA The Hague, The Netherlands

**Keywords:** Forensic anthropology, Cranial vault thickness, Biological profile, Age, Sex, Ancestry

## Abstract

The relation between human cranial vault thickness (CVT) and various elements of the physical anthropological biological profile is subject of ongoing discussion. Some results seem to indicate no correlation between CVT and the biological profile of the individual, whereas other results suggest that CVT measurements might be useful for identification purposes. This study assesses the correlation between CVT and body weight, stature, age, sex, and ancestry by reviewing data of 1097 forensic autopsies performed at the Netherlands Forensic Institute (NFI). In subadults (younger than 19 years of age at the time of death), all frontal, temporal, and occipital CVT measurements correlated moderately to strongly with indicators of growth (body weight, stature, and age). Neither sex nor ancestry correlated significantly with cranial thickness. In adults, body weight correlated with all CVT measurements. No meaningful correlation was found between CVT and stature or age. Females showed to have thicker frontal bones, and the occipital region was thicker in the Negroid subsample. All correlation in the adult group was weak, with the distribution of cranial thickness overlapping for a great deal between the groups. Based on these results, it was concluded that CVT generally cannot be used as an indicator for any part of the biological profile.

## Introduction

The skull is one of most studied elements of the human skeleton. Its morphological features in relation to sex, age, and ancestry have identified it as one of the most useful sources of information for the compilation of a biological profile of an unknown individual, e.g., [1, 2].

Several attempts have been made to investigate the association between cranial vault thickness (CVT) and sex, age at the time of death, and/or ancestry, with conflicting results. Some authors reported a significant difference in CVT between males and females [3–7] whereas others did not [8–14]. The same holds true for results regarding correlations with age [3, 9–12, 14–18] and ancestry [3–5, 12]. Despite these inconsistencies, it is still suggested that the age, sex, and body mass index of an unknown individual can be inferred from CVT measurement [7, 17].

A substantial part of the research on cranial vault thickness was performed on small sample populations, often affected by selection bias. Also, variation in sampling methods or sampling topography hampers meta-analysis of the data. As a result, little is known regarding the normal variation of CVT within population groups, and there is a lack of robust evidence to support or refute a possible correlation between CVT and the various variables of the anthropological biological profile.

Two illustrative Dutch cases emphasize the importance of such knowledge in forensic anthropological casework. In 1939, a female passed away due to complications of external mechanical blunt head trauma delivered by her husband who threw a slipper at her head (Court Amsterdam, 14-06-1939, NJ 1940, 34). In 1950, a similar case presented itself, when a man passed away after a blow to the head with a copper tube (Dutch Supreme Court, 24-01-1950, NJ 1950, 293). In both cases, forensic autopsy demonstrated an extraordinary thin cranial vault. Expert forensic anthropological assistance was requested with the examination focusing on CVT and its possible relation to increased fracture risk. The occurrence of several similar cases over the past 50 years endorses the need for knowledge on natural variation of cranial vault thickness.

The aims of this study are twofold. First, it aims to report the variation in CVT in a contemporary forensic population. Second, it aims to provide insight into the relation between CVT and body weight, stature, age, sex, and ancestry.

## Material and methods

The CVT measurements of all forensic autopsies performed at the Netherlands Forensic Institute (NFI) in the period January 2011 to March 2014 were retrospectively reviewed.

Measurement of CVT is part of the standard procedure in every forensic autopsy at the NFI. The scalp is incised and pulled anteriorly exposing the calvarium. The calvarium is removed by a transverse cut that bisects the left and right euryon and the opistocranion. After removal of the brain, full CVT is measured to the nearest millimeter with a ruler at three locations: Frontal cranial thickness (FCT) is measured just lateral to the crista frontalis, lateral cranial thickness (LCT) at either the right or left euryon, and occipital cranial thickness (OCT) at the opistocranion.

Postmortem body weight, postmortem body stature, age at death, and sex was retrieved from the autopsy reports. Cases of head trauma that hampered correct measurements or cases with missing data or obviously flawed measurements (e.g., body weight in a severely burned individual) were excluded from the study, resulting in a total of 1097 cases. None of the cases presented with pathology that interfered with CVT.

The documentation of ancestral origin is not a part of the standard operating procedure at the NFI and was thus designated by reviewing facial photographs. Photographical designation of ancestral origin will generally concur with the ancestral group to which the individual was perceived to be a member while living [19]. Due to overlapping phenotypes, this approach dictates relatively broad ancestral groups (Caucasian, Negroid, or Mongoloid).

Of all measurements, means and standard deviations were calculated. The degree of correlation between CVT measurements and continuous variables (other CVT measurements, body weight, stature, and age) was evaluated with Pearson’s correlation coefficient (*R*). Significant correlation required a *p* value of <0.05. R-values were interpreted as proposed by Zhou et al. [20] with *R* values approximating −0.2/0.2, −0.5/0.5, −0.8/0.8, and −1/1 indicating weak, moderate, strong, and perfect correlation respectively.

Association between the cranial thickness measurements and the categorical variables (sex and ancestry) was evaluated with a one-way analysis of variance (ANOVA). When the *p* value of the ANOVA test was significant, i.e., less than 0.05, Tukey’s post hoc analysis was performed to investigate the intergroup differences.

Individuals younger than 19 years of age were classified as subadults (*n* = 103). The adult group consisted of 994 individuals. In order to investigate a possible difference in CVT development between subadult males and females, the means of the three CVT measurements as observed at age 10 to 19 years were compared between the sexes by means of an independent samples *t* test. All statistical analyses were performed with IBM SPSS Statistics 21.

The demographic composition of the sample population can be seen in Tables 1 and 2. When considering the adults included in this study, the Negroid subsample was significantly younger when compared to the Mongoloid and Caucasoid groups. The Mongoloid subgroup was significantly shorter and weighed less than the other two groups. The adult females were significantly older and shorter and weighed less than the male group.

**Table 1:** Demographic information of the subadult sample

	Male			Female		
N	61			42		
Ancestry	Caucasoid		47 (78 %)	Caucasian		34 (81 %)
	Negroid		8 (13 %)	Negroid		3 (7 %)
	Mongoloid		4 (6 %)	Mongolian		2 (5 %)
	Indifferent		2 (3 %)	Indifferent		3 (7 %)
Age	*N*	Weight (SD)	Stature (SD)	*N*	Weight (SD)	Stature (SD)
<6	34	9.48 (6.28)	72.72 (18.25)	20	8.40 (4.79)	57.29 (8.16)
6-11	10	31.10 (6.19)	136.90 (9.13)	8	41.50 (12.38)	146.63 (14.08)
12-19	17	66. 52 (15.33)	173.64 (15.27)	14	64.00 (12.02)	166.86 7.41)

Age is in years, stature in centimeters, and weight in kilograms

*N* number of individuals, *SD* standard deviation

**Table 2:** Demographic information of the adult sample

	N	Age (SD)	Stature (SD)	Weight (SD)	♂/♀
Total sample					
Males	655	43.13 (15.74)	178.85 (8.89)	83.70 (15.75)	
Females	339	49.82 (18.88)	165.77 (8.26)*	70.70 (18.66)*	
Ancestral groups					
Caucasoid	792	46.39 (17.14)	174.58 (10.73)	79.75 (20.59)	514/278
Negroid	111	38.60 (13.44)^†^	175.59 (9.39)	81.03 (8.13)	85/26
Mongoloid	36	41.55 (18.86)	168.28 (10.79)^†^	71.40 (17.64)^†^	19/17
Indifferent	55				

Age is in years, stature in centimeters, and weight in kilograms

*N* number of individuals, *SD* standard deviation

*Significantly different from the male group, *p* < 0.001. †Significantly different from the other two ancestral groups (one-way ANOVA, post hoc Tukey test) with *p* < 0.001

## Results

**Fig. 1:**
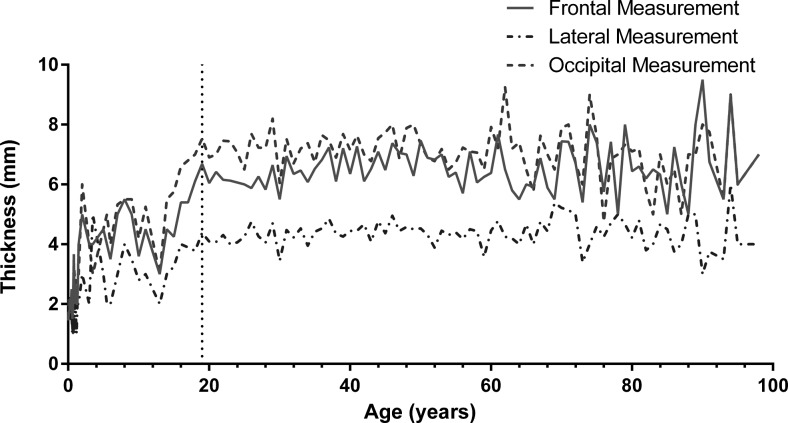
Mean frontal, temporal, and occipital cranial vault thickness measurements plotted against age (*n* = 1097). The *dashed vertical line* at age 19 divides the subadult and adult subsamples

Skull thickness appears to increase until approximately 19 years of age (see Fig. 1). The CVT measurements at the three different locations correlated positively with one another (*R* values between 0.521 and 0.558, *p* < 0.001).

## Subadults

**Fig. 2:**
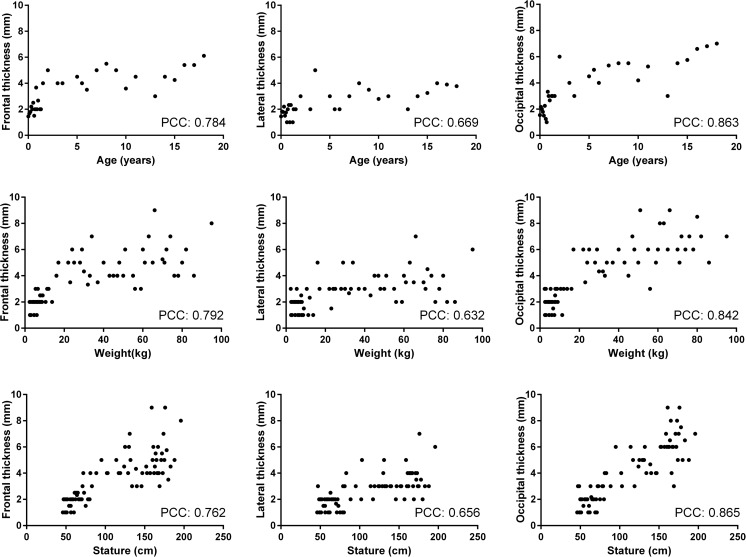
Mean cranial thickness measurements plotted for age, weight, and stature in the subadult sample. PCC = Pearson’s correlation coefficient

In the subadult group (*n* = 103), the independent samples *t* test did not show any significant difference between males and females between age 10 to 19 (*p* values between 0.148 and 0.907). CVT correlated positively with body weight, stature, and age (*R* values between 0.632 and 0.865, *p* < 0.001. See Fig. 2). One-way ANOVA analysis identified no association between CVT measurements and sex or ancestry, except for the occipital measurement in Mongoloid subadults (see Table 3). However, this association is probably the result of the skewed Mongoloid subadult sample composition. The Mongoloid group consisted of only six individuals, all older than 14 years of age at the time of death.

**Table 3:** Mean frontal, lateral, and occipital cranial thickness for sex and ancestral groups in the subadult sample

	FCT (SD)	LCT (SD)	OCT (SD)
Sex			
Male (*n* = 61)	3.67 (1.76)	2.67 (1.26)	4.02 (2.06)
Female (*n* = 42)	3.45 (1.97)	2.57 (1.25)	4.06 (2.52)
Ancestry			
Caucasoid (*n* = 82)	3.59 (1.85)	2.65 (1.27)	3.99 (2.24)
Negroid (*n* = 10)	2.80 (1.31)	2.30 (1.16)	3.20 (1.55)
Mongoloid (*n* = 6)	5.00 (0.89)	3.50 (0.84)	6.83 (1.42)*
Indifferent (*n* = 5)	2.80 (2.68)	1.60 (0.89)	2.40 (1.67)

*N* number of individuals, *SD* standard deviation, *FCT* frontal cranial thickness, *LCT* lateral cranial thickness, *OCT* occipital cranial thickness

*Significantly different from the other two ancestral groups (one-way ANOVA, post hoc Tukey test) with *p* < 0.012

## Adults

**Fig. 3:**
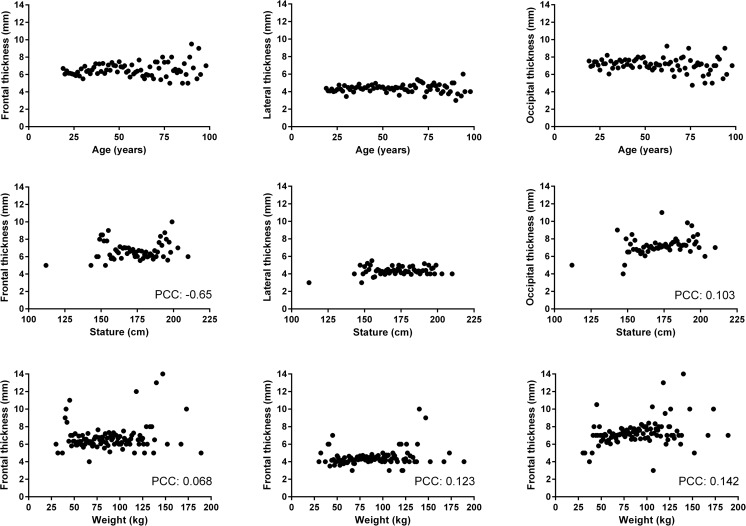
Mean cranial thickness measurements plotted against age, body weight, and stature in the adult sample. PCC = Pearson’s correlation coefficient. Only significant PCCs (*p* < 0.05) are given

In adults, body weight showed a significant, yet very weak correlation with all cranial thickness measurements (*R* = 0.068–0.142, *p* < 0.033, see Fig. 3). Correlation with stature was ambiguous. Correlation between stature and FCT was negative (*R* = −0.065, *p* = 0.041), whereas correlation with OCT was positive (*R* = 0.103, *p* = 0.001). No significant correlation existed between stature and LCT (*p* = 0.512). No significant correlation was found between cranial thickness and age (*p* = 0.112–0.188).

Since the male and female samples differed significantly with respect to body weight, stature, and age, all the correlation analyses were repeated for males and females separately. Body weight still correlated weakly positive with all cranial thickness measurements (*R* between 0.201 and 0.095, *p* < 0.016). Correlation between stature and CVT now showed to be non-significant, except for FCT in males, which presented with a significant, yet very weak positive correlation (*R* = 0.088, *p* = 0.026). No significant correlation could be demonstrated between age and the cranial thickness measurements for either males or females (*p* = 0.109–0.808).

The means of the CVT measurements, grouped by sex and ancestry, are shown in Table 4. A significant difference was observed for FCT and OCT between males and females. Males presented with a thinner FCT (6.15 vs. 7.13 mm, *p* < 0.001), but thicker OCT (7.33 vs. 6.89 mm, *p* < 0.001). When corrected for body weight, the difference in mean FCT became larger (6.09 vs. 7.26 mm, *p* < 0.001), whereas the difference in mean OCT became non-significant (7.28 vs. 7.00 mm, *p* = 0.093).

**Table 4:** Mean frontal, lateral, and occipital cranial thickness for sex and ancestral groups in the adult sample

	FCT (SD)	LCT (SD)	OCT (SD)
Sex			
Male (*n* = 655)	6.15 (1.91)	4.33 (1.22)	7.33 (2.01)
Female (*n* = 339)	7.13 (2.30)*	4.41 (1.15)	6.89 (2.00)*
Ancestry			
Caucasoid (*n* = 792)	6.47 (2.10)	4.35 (1.20)	7.08 (1.92)
Negroid (*n* = 111)	6.77 (2.26)	4.39 (1.21)	8.05 (2.40) ^†^
Mongoloid (*n* = 36)	5.97 (1.59)	4.08 (0.69)	6.72 (1.68)
Indifferent( *n* = 55)	6.50 (1.96)	4.55 (1.28)	7.38 (2.24)

*N* number of individuals, *FCT* frontal cranial thickness, *LCT* lateral cranial thickness, *OCT* occipital cranial thickness.

*Significantly different from the male group, *p* < 0.001. † Significantly different from the two other ancestral groups (one-way ANOVA post hoc Tukey tests) with *p* < 0.003

To determine whether the difference in FCT is related to ageing, the CVT measurements of males and females younger and older than 55 years were compared. Mean FCT was significantly higher in younger males (6.30 mm (younger males) vs. 5.78 mm (older males), *p* = 0.006); there was no difference between younger and older females (7.16 vs. 7.21 mm, *p* = 0.820).

There was no significant difference in CVT measurements between the ancestral groups, except for OCT in the Negroid group (*p* < 0.003). Correction for body weight did not alter this result. Also, a separate analysis of males and females within the ancestral groups was generally without effect; only the difference in OCT between Negroid and Mongoloid males became non-significant (*p* = 0.247). CVT measurements and age did not correlate significantly in the ancestral groups (*p* values between 0.158 and 0.928).

## Discussion

In the subadult group, all cranial measurements correlated strongly with body weight, stature, and age. These observations are in line with earlier publications [3, 18, 21]. Our results support that growth is one of the most important factors in CVT development. The increase of CVT levels offs at the end of adolescence and thus seems to end around the same time as brain volume development and postcranial skeletal growth. Brain volume is known to reach its peak around 19–20 years of age [21], and most epiphyseal plates, especially those of the long bones, are closing or closed at this time [2].

The available literature on the correlation between sex and CVT in subadults is very limited. Our results corroborate the findings of a recent, but smaller study that reported no differences in cranial vault thickness and the sexes in 72 subadults [14]. As such, CVT development does not appear to show the sexual dimorphism that is seen in postcranial skeletal development. The exact mechanism by which growth affects cranial thickness remains unclear, but a multivariable theory, with systemic factors (growth hormones, exercise) and local factors (development of the brain and muscle strength) seems most likely [14].

To our knowledge, our survey is the first to report on the association between CVT and ancestry in subadults. Our results did not identify an association, but it must be kept in mind that the Negroid and Mongloid ancestral groups were of insufficient size to infer any robust conclusions.

In the adult group, the positive correlation between all CVT measurements and body weight implicates that general body build has an effect on cranial thickness. It is well known that activity-induced strain has a localized osteoblastic effect. With the strongest correlation found in the occipital region, where the most muscle attachments are located, this also seems to hold true for the bones of the. As such, our results are in line with earlier suggestions that muscle activity is at least one of the factors responsible for cranial vault thickness [14, 22].

Our initial analysis identified a difference in OCT between males and females, which seemed to corroborate earlier reports [7]. However, our male group was significantly heavier than the female sample, and thus our initial finding probably underestimated OCT in females. When correcting for the confounding effect of body weight, indeed the difference became non-significant.

This study is not the first to report a significant difference in frontal bone thickness between males and females. Earlier studies with similar findings adopted hyperostosis frontalis interna (HFI) as a possible explanation [7, 9]. HFI is a localized and benign thickening of the inner side of the frontal bone, is mainly seen in older women, and is believed to increase gradually with age [23, 24]. The absent correlation between FCT and age, especially in the female sample, seems to argue against HFI as an explanation. The same holds for the lack of a significant difference in FCT between younger and older females. Since HFI is generally less pronounced along the midline [23] and is generally more pronounced in older females [24], our measurement point and relatively young study population might be less sensitive to HFI-related increase in FCT. Whether the difference in FCT between males and females can be attributed to HFI therefore remains open.

Naturally, body weight and stature are positively correlated, and one would thus expect the correlations between CVT and body weight or stature to be similar. However, the results proved ambiguous. The negative correlation between FCT and stature might be partially explained by the difference in FCT between males and females, since females were significantly shorter. This is supported by the separate analysis of males and females in which the correlation becomes non-significant for females and less significant for males. Also, the initial (weak) positive correlation between stature and OCT might be affected by confounding, since the male group was significantly larger and heavier. Indeed, when females and males were analyzed separately, this correlation is no longer significant.

The correlation between age and cranial thickness has been extensively studied. Many studies suggested an association between age and cranial thickness in adults [3, 4, 9, 13, 15, 17, 18], whereas others did not [10–12, 14, 17]. Our data did not show any correlation between CVT and age in adults. Further analyses separated by sex and ancestry did not alter this result. Our study therefore supports the latter, more recent publications.

In general, our results did not identify an association between ancestry and CVT. Only the Negroid subgroup had a higher mean OCT than the two other groups. Separate analysis of males and females generally did not alter this results, expect for the difference in OCT between Negroid and Mongoloid males. The value of this observation is however limited due to the small size of the Mongoloid male subgroup. The Negroid subgroup was slightly heavier, but correction for body weight had no effect. The exact cause of the difference in OCT between the Negroid subsample and the other ancestral subgroups remains open for discussion.

For the adult group, any correlation between CVT measurements and the continuous variables was (very) weak. This makes it impossible to reliably deduce body weight, stature, or age from CVT measurement. The differences between the reported means in the categorical values are in the range of 1 to 2 mm. With standard deviations of a similar range, it becomes apparent that a large deal of overlap exists in cranial thickness between the sexes and ancestral groups. We therefore endorse the suggestion of Lynnerup who stated that the inherent high variation in cranial vault thickness obstructs any significant correlation to age, sex, or ancestry [10]. We conclude that it is generally impossible to make meaningful assumptions on any element of the forensic anthropological biological profile from cranial vault thickness measurement.

## Conclusion

Our retrospective survey of 1097 Dutch forensic autopsy cases show that in individuals younger than 19 years, cranial thickness correlates strongly with continuous variables of growth (stature, weight, and age). In adults, weight shows a weakly positive correlation with all cranial thickness measurements. Females appear to have a larger frontal cranial thickness, whereas the Negroid subsample demonstrated a larger occipital cranial thickness. The other parameters included in this study did not correlate with cranial vault thickness measurements. As all correlations observed were (very) weak, and there was a large deal of overlap between the various subgroups, cranial thickness measurement cannot be used as a proxy for elements of the forensic anthropological biological profile.
